# Influencing factors on morbidity and mortality in intertrochanteric fractures

**DOI:** 10.1038/s41598-023-38667-9

**Published:** 2023-07-26

**Authors:** Mazyar Babagoli, Amirhossein Ghaseminejad Raeini, Mehrdad Sheykhvatan, Soroush Baghdadi, Seyyed Hossein Shafiei

**Affiliations:** 1 Sina University Hospital, Tehran University of Medical Sciences, Tehran, Iran; 2 Orthopedic Surgery Research Center, Sina University Hospital, Tehran University of Medical Sciences, Tehran, Iran; 3 Pediatric Orthopaedic Surgery Department, Montefiore Medical Center, New York, USA

**Keywords:** Trauma, Outcomes research

## Abstract

We aimed to evaluate the effect of the patient’s clinical and paraclinical condition before and after surgery on short-term mortality and complication and long-term mortality. A retrospective cohort study was conducted and multivariate logistic regression was applied to determine the effect of demographic characteristics (sex, age, AO/OTA classification, height, weight, body mass index), medical history (hypertension, ischemic heart disease, diabetes mellitus, thyroid malfunction, cancer, osteoporosis, smoking) lab data (Complete blood cell, blood sugar, Blood Urea Nitrogen, Creatinine, Na, and K), surgery-related factors (Anesthesia time and type, implant, intraoperative blood transfusion, postoperative blood transfusion, and operation time), duration of admission to surgery and anticoagulant consumption on short-term mortality and complication and long-term mortality. Three hundred ten patients from November 2016 to September 2020 were diagnosed with an intertrochanteric fracture. 3.23% of patients died in hospital, 14.1% of patients confronted in-hospital complications, and 38.3% died after discharge till the study endpoint. ΔNumber of Neutrophiles is the primary determinant for in-hospital mortality in multivariate analysis. Age and blood transfusion are the main determinants of long-term mortality, and Na before surgery is the primary variable associated with postoperative complications. Among different analytical factors Na before surgery as a biomarker presenting dehydration was the main prognostic factor for in hospital complications. In hospital mortality was mainly because of infection and long-term mortality was associated with blood transfusion.

## Introduction

In an aging population, osteoporotic fractures continue to rise. By the year 2025, there are estimated to be 2.6 million hip fractures, which will increase to 4.5 million by 2050. The changes will be more substantial in Asia, with the percentage of fragility fractures is estimated to increase from 26% of all hip fractures in 1990 to 37% in 2025, and to 45% in 2050^1^. The estimated cost of intertrochanteric hip fractures in the United States healthcare system is $2.63 billion USD per year which represents 44% of all hip fracture costs^2^. Apart from the economic burden, the risks of subsequent fracture following a hip fracture, mortality, and morbidity including impaired mobility and decreased quality of life remain considerable compared to the general population^3^.

The 1-year mortality rate for intertrochanteric fractures has decreased from 34 to 23% gradually in the literature^4^. Still, in-hospital complications and mortality following hip fracture is reported in up to 13% and 5%, respectively^5^. Associations have been found between the surgical approach^6^, Charlson Comorbidity Index^7^, delay from admission to surgery^8^, body mass index^9^, anticoagulant consumption^10–12^, type of anesthesia^13^ and mortality or complications following hip fractures surgery have been previously discussed. Pre-operative factors like anemia^14^, nutritional parameters^15^, analytical values^16^, blood parameters^17^, and neutrophile to lymphocyte ratio can be measured by a blood test and the prognostic role of these variables is valuable.

We aimed to evaluate the risk factors for morbidity/mortality in patients undergoing surgery for a hip fracture. In particular, we were interested in the association between demographic characteristics, AO/OTA fracture classification, comorbidities, surgical variables, and laboratory tests before and after surgery with in-hospital complications, in-hospital mortality and long-term mortality.

## Material and methods

### Study population

We retrospectively reviewed data from all consecutive patients admitted with intertrochanteric fractures to a trauma referral center Tehran, Iran, from November 2016 to September 2020. Bilateral, high-energy, peri-prosthetic, and pathologic fracture, as well as non-operatively treated patients were excluded. Conservative treatment is only indicated for patients who do not agree to undergo surgery. Institutional IRB approval was obtained prior to data collection. All participants gave informed consent, and the proposal was approved by the Tehran University of Medical Sciences review board. All methods were performed in accordance with the approved guidelines and regulations of Tehran University of Medical Sciences.

### Data collection

Charts were reviewed to collect the patients’ demographic information (age, height, weight, body mass index (BMI)), past medical history, medication history, substance use, and family history. Lab results of interest were also collected, including cell count, biochemistry, and metabolic profile. Radiographs at the time of admission were reviewed to classify the fracture type based on the AO/OTA classification. Surgical variables including surgical and anesthesia time, type of surgery and anesthesia, and blood transfusion were also collected.

ΔVariable was defined as:

$$\Delta {\text{Variable }} = {\text{ Variable on post-operation day 1}}{-}{\text{Variable before surgery}}.$$

### Outcomes

In November 2021, patients were contacted by phone. To determine the outcome, patient or close family were asked whether the patient was alive and, if not, the passing date. Mortality in hospital (post-operative) and complications were obtained from charts. All outcomes are expressed as a binary variable.

### Statistical analysis

SPSS version 23 for windows (IBM, Armonk, New York) was used for the statistical analysis Data are presented as the mean and standard deviation or the number of cases and percentages whenever needed. Student’s *t* test, Pearson, chi-square, and Fisher exact tests were used as appropriate to assess unadjusted associations between variables and outcomes. A p-value of <0.1 was considered significant for univariate analysis. Hosmer-Lemeshow test was performed to evaluate our final regression model. Furthermore, we conducted ROC analysis to develop a screening test. A p-value of <0.05 was considered significant for binary logistic regression, cox regression, and Kaplan-Meier analysis. A p-value of <0.05 was considered significant for ROC analysis and Area under the curve (AUC) > 0.70 was acceptable to achieve a screening test with maximum sensitivity and specificity. The Youden J statistic was applied to determine optimal cutoff points^18^. Our statistical analysis was carried out in consultation with a statistician.

### Ethics approval and consent to participate

The ethics committee of Tehran University of Medical Sciences, Tehran, Iran, has approved. This manuscript. Written informed consent was obtained from patients for publication and all participants gave their consent for participation.

## Results

During the study period Three hundred ten patients were diagnosed with an intertrochanteric fracture. 270 patients had full-recorded progress notes in which in hospital complication could be assessed (87%). 67 patients lost to follow-up, which results in a sample size of 243 patients for mortality in long term (81%). All post operative complications are presented on Table 1. The percentage of the female population in those who died in hospital, had complications in hospital, and died in log-term after discharge are as follows: 30%, 34.2%, and 59.1%; Also the mean age of those who died in hospital, had complications in hospital, and died in log-term after discharge is 82.30, 76.37, and 78.55 years respectively. 3.23% of patients died in hospital, 14.1% of patient confront in hospital complication and 38.3% died after discharge till study endpoint. Patients’ data are shown in Table 2.

**Table 1:** The number of patients who experienced post-operative complications.

Postoperative complication	Number of patients
Cardiac complication	10
Diaphoresis	1
Surgical site discharge	6
Bed sore	2
Surgical site bleeding	7
Surgical site infection	1
Pulmonary embolism	2
Unbearable pain	1
Sensory motor disturbance	1
Gastrointestinal bleeding	1
Deep vein thrombosis	1
Hyponatremia	1
Sepsis	2
ARDS	1
Agitation	2

**Table 2:** Demographic characteristics, lab data, surgical technique, and outcomes of patients.

Characteristic	In-hospital mortality (No. (%) of patients, (n=missing))		P-value	Long term mortality (No. (%) of patients, (n=missing))		P-value	In-hospital complication (No. (%) of patients, (n=missing))		P-value
	Yes=10	No=300		Yes=93	No=150		Yes=38	No=232	
Sex			0.207			**0.013**			0.107
Female	3 (30%), (n=0)	145 (48.3%), (n=0)	–	55 (59.1%), (n=0)	62 (41.3%), (n=0)	–	13 (34.2%), (n=0)	112 (48.3%), (n=0)	–
Age (yr) *	82.30 ± 8.69 (n=0)	71.46 ± 14.96 (n=0)	**0.003**	78.55 ± 9.35 (n=0)	66.55 ± 17.01 (n=0)	**0.000**	76.37 ± 11.38 (n=0)	70.22 ± 15.77 (n=0)	**0.022**
AO/OTA	(n=1)	(n=45)	0.718	(n=15)	(n=21)	0.451	(n=3)	(n=35)	0.979
31A1.2	3 (33.3%)	119 (46.7%)	–	31 (39.7%)	67 (51.9%)	–	15 (42.9%)	91 (46.2%)	–
31A1.3	4 (44.4%)	59 (23.1%)	–	20 (25.6%)	27 (20.9%)	–	9 (25.7%)	47 (23.9%)	–
31A2.2	1 (11.1%)	41 (16.1%)	–	17 (21.8%)	17 (13.2%)	–	7 (20.0%)	30 (15.2%)	–
31A2.3	1 (11.1%)	12 (4.7%)	–	5 (6.4%)	5 (3.9%)	–	1 (2.9%)	11 (5.6%)	–
31A3.1	0 (0%)	8 (3.1%)	–	2 (2.6%)	4 (3.1%)	–	1 (2.9%)	6 (3.0%)	–
31A3.2	0 (0%)	3 (1.2%)	–	2 (2.6%)	0 (0%)	–	0 (0%)	1(.5%)	–
31A3.3	0 (0%)	13 (5.1%)	–	1 (1.3%)	9 (7.0%)	–	2 (5.7%)	11 (5.6%)	–
Smoking	3 (30%), (n=0)	76 (26%), (n=8)	0.725	14 (15.2%), (n=1)	49 (33.1%), (n=2)	**0.008**	9 (23.7%), (n=0)	65 (28.4%), (n=3)	0.549
Height (cm)*	166.80 ± 8.95 (n=0)	165.82 ± 9.61 (n=45)	0.741	163.64 ± 8.56 (n=8)	167.19 ± 10.00 (n=24)	**0.012**	167.94 ± 8.08 (n=5)	166.16 ± 9.65 (n=29)	0.316
Weight (Kg)*	70.56 ± 18.24 (n=1)	69.14 ± 12.09 (n=43)	0.736	67.91 ± 13.35 (n=7)	70.37 ± 11.27 (n=23)	0.130	69.56 ± 15.42 (n=6)	70.00 ± 11.80 (n=27)	0.880
BMI (Kg/m^2^)*	25.14 ± 4.84 (n=1)	25.13 ± 4.07 (n=35)	0.995	25.34 ± 4.60 (n=7)	25.13 ± 3.55 (n=16)	0.760	24.51 ± 3.98 (n=5)	25.36 ± 4.19 (n=21)	0.259
Duration of admission to surgery (day)*	6.80 ± 6.36 (n=0)	5.63 ± 3.66 (n=0)	0.335	6.52 ± 4.06 (n=0)	5.25 ± 3.46 (n=0)	**0.004**	5.97 ± 5.27 (n=0)	5.76 ± 3.50 (n=0)	0.751
Anticoagulant consumption	4 (44.4%), (n=1)	106 (36.3%), (n=8)	0.729	37 (40.2%), (n=1)	46 (31.7%), (n=5)	0.110	16 (43.2%), (n=1)	79 (35%), (n=6)	0.331
Past medical history									
HTN (hypertension)	8 (80%), (n=0)	136 (45.6%), (n=2)	**0.050**	49 (53.3%), (n=1)	54 (36%), (n=0)	**0.012**	23 (60.5%), (n=0)	104 (45%), (n=1)	**0.076**
IHD (Ischemic heart disease)	6 (60%), (n=0)	67 (22.6%), (n=3)	**0.014**	26 (28.6%), (n=2)	27 (18%), (n=0)	**0.051**	14 (36.8%), (n=0)	53 (23%), (n=2)	**0.069**
DM (Diabetes mellitus)	2 (20%), (n=0)	82 (27.6%), (n=3)	0.733	35 (38.5%), (n=2)	29 (19.3%), (n=0)	**0.004**	9 (23.7%), (n=0)	65 (28.3%), (n=2)	0.559
Thyroid malfunction	0 (0%), (n=0)	24 (8.1%), (n=2)	1.000	6 (6.5%), (n=1)	12 (8%), (n=0)	0.748	1 (2.6%), (n=0)	20 (8.7%), (n=1)	0.327
Cancer	0 (0%), (n=6)	9 (4%), (n=74)	1.000	5 (6.5%), (n=16)	4 (2.7%), (n=3)	0.104	1 (3.8%), (n=12)	7 (4%), (n=57)	1.000
Osteoporosis	1 (25%), (n=6)	40 (18.3%), (n=81)	0.559	17 (23%), (n=19)	23 (16.1%), (n=0)	0.340	6 (23.1%), (n=12)	31 (18.2%), (n=62)	0.592
Lab data									
Hemoglobin before surgery (mg/dL)*	10.62 ± 1.85 (n=0)	11.69 ± 1.87 (n=4)	0.103	10.92 ± 1.52 (n=0)	12.23 ± 1.84 (n=4)	**0.000**	11.18 ± 2.11 (n=0)	11.78 ±1.84 (n=3)	0.107
Δ Hemoglobin (mg/dL)*	− 1.73 ± 1.79 (n=0)	− 1.35 ± 1.93 (n=88)	0.527	− 1.00 ± 1.83 (n=17)	− 1.52 ± 1.97 (n=57)	**0.020**	− 1.77 ± 1.79 (n=3)	− 1.27 ± 1.93 (n=70)	0.147
Blood sugar before surgery (mg/dL)*	151.22 ± 64.34 (n=1)	143.23 ± 53.75 (n=52)	0.722	151.48 ± 57.78 (n=11)	136.77 ± 47.71 (n=29)	0.193	138.68 ± 49.53 (n=4)	143.10 ± 54.83 (n=42)	0.639
Δ Blood sugar (mg/dL)*	− 20.22 ± 43.30 (n=1)	− 4.64 ± 62.70 (n=157)	0.333	− 3.42 ± 70.41 (n=33)	− 9.04 ± 61.8 (n=93)	0.259	− -15.37 ± 64.91 (n=11)	− 2.30 ± 63.16 (n=127)	0.354
White blood cell before surgery (×10^3^)*	11.59 ± 4.04 (n=1)	9.34 ± 2.89 (n=8)	**0.024**	9.42 ± 3.15 (n=1)	9.32 ± 2.80 (n=6)	0.787	9.51 ± 2.83 (n=2)	9.39 ± 3.04 (n=6)	0.816
Δ White blood cell (×10^3^)*	4.63 ± 6.61 (n=1)	1.63 ± 3.96 (n=102)	**0.033**	2.14 ± 4.02 (n=21)	1.67 ± 3.94 (n=66)	0.470	1.95 ± 3.39 (n=5)	1.63 ± 4.23 (n=83)	0.640
Platelet before surgery (×10^3^)*	235.70 ± 78.68 (n=0)	226.41 ± 86.32 (n=8)	0.722	230.95 ± 84.38 (n=1)	225.55 ± 82.17 (n=6)	0.709	232.38 ± 89.97 (n=1)	226.59 ± 85.23 (n=6)	0.716
Δ Platelet (×10^3^)*	− 25.80 ± 57.98 (n=0)	25.86 ± 65.55 (n=102)	**0.021**	22.78 ± 68.16 (n=21)	21.92 ± 59.15 (n=66)	0.690	21.68 ± 63.24 (n=4)	26.65 ± 66.16 (n=83)	0.683
% Neutrophile before surgery*	71.74 ± 12.85 (n=0)	74.61 ± 9.15 (n=12)	0.501	74.37 ± 9.00 (n=3)	73.99 ± 9.67 (n=6)	0.569	72.37 ± 10.46 (n=1)	74.69 ± 9.31 (n=9)	0.211
Δ% Neutrophile*	6.47 ± 10.00 (n=0)	3.85 ± 10.70 (n=109)	0.439	5.85 ± 10.62 (n=22)	3.78 ± 10.54 (n=70)	0.330	4.47 ± 11.27 (n=4)	3.70 ± 10.59 (n=89)	0.717
% Lymphocyte before surgery*	17.40 ± 13.18 (n=0)	16.19 ± 7.25 (n=7)	0.617	16.29 ± 6.94 (n=1)	16.99 ± 7.89 (n=4)	0.400	17.56 ± 8.67 (n=1)	16.33 ± 7.50 (n=5)	0.416
Δ% Lymphocyte*	− 3.69 ± 8.10 (n=0)	− 4.19 ± 7.67 (n=104)	0.853	− 5.02 ± 6.99 (n=20)	− 4.59 ± 8.23 (n=67)	0.846	− 5.20 ± 7.83 (n=5)	− 3.94 ± 7.49 (n=84)	0.406
Cr before surgery (mg/dL)*	1.80 ± 0.62 (n=0)	1.20 ± 0.59 (n=23)	**0.013**	1.31 ± 0.74 (n=4)	1.07 ± 0.26	**0.000**	1.39 ± 0.64 (n=4)	1.19 ± 0.60 (n=18)	**0.100**
Δ Cr (mg/dL)*	0.13 ± 0.27 (n=0)	− 0.05 ±0.37 (n=145)	**0.077**	− 0.04 ± 0.53	− 0.06 ± 0.18 (n=91)	0.907	0.12 ± 0.36 (n=9)	− 0.07 ± 0.38 (n=116)	**0.017**
BUN before surgery (mg/dL)*	87.10 ± 40.66 (n=0)	51.06 ± 27.71 (n=24)	**0.000**	60.16 ± 33.68 (n=4)	42.53 ± 16.11 (n=14)	**0.000**	56.74 ± 30.71 (n=4)	50.77 ± 28.33 (n=19)	**0.**294
Δ BUN (mg/dL)*	12.60 ± 33.39 (n=0)	3.86 ± 24.81 (n=145)	0.435	5.88 ± 32.17 (n=30)	− 0.017 ± 15.61 (n=90)	0.658	12.52 ± 26.97 (n=9)	3.17 ± 25.25 (n=117)	**0.099**
Na before surgery (mg/dL)*	138.03 ± 4.15 (n=0)	137.72 ± 7.35 (n=40)	0.826	137.96 ± 4.17 (n=6)	137.49 ± 9.73 (n=27)	0.765	139.58 ± 4.41 (n=4)	137.38 ± 8.02 (n=32)	**0.022**
Δ Na (mg/dL)*	2.58 ± 6.43 (n=0)	0.36 ± 5.01 (n=151)	0.312	1.05 ± 5.94 (n=31)	− 0.76 ± 4.42 (n=94)	**0.064**	− 0.35 ± 7.18 (n=10)	0.50 ± 4.72 (n=121)	0.557
K before surgery (mg/dL)*	4.26 ± 0.60 (n=0)	4.20 ± 0.46 (n=40)	0.790	4.28 ± 0.49 (n=5)	4.14 ± 0.41 (n=27)	**0.076**	4.21 ± 0.41 (n=4)	4.19 ± 0.48 (n=31)	0.821
Δ K (mg/dL)*	0.20 ± 0.65 (n=0)	0.31 ± 0.64 (n=151)	0.640	0.32 ± 0.69 (n=31)	0.30 ± 0.56 (n=94)	0.433	0.34 ± 0.65 (n=10)	0.30 ± 0.62 (n=121)	0.753
Neutrophile/platelet before surgery*	0.039 ± 0.010 (n=1)	0.035 ± 0.016 (n=14)	0.200	0.034 ± 0.016 (n=3)	0.034 ± 0.016 (n=8)	0.836	0.033 ± 0.013 (n=2)	0.035 ± 0.016 (n=11)	0.624
Δ Neutrophile/platelet*	0.04237 ± 0.062 (n=1)	0.00336±0.01759 (n=109)	**0.000**	0.00507±0.01584 (n=22)	0.00433 ± 0.01451 (n=70)	0.650	0.00708 ± 0.01979 (n=5)	0.00376 ± 0.02275 (n=89)	0.403
Neutrophile/lymphocyte before surgery*	6.48 ± 2.71 (n=1)	5.78 ± 3.09 (n=13)	0.462	5.52 ± 2.75 (n=3)	5.68 ± 3.29 (n=6)	0.759	5.58 ± 3.49 (n=2)	5.78 ± 3.09 (n=10)	0.748
Δ Neutrophile/lymphocyte *	5.41 ± 7.41 (n=1)	2.73 ± 4.97 (n=111)	0.125	3.78 ± 4.89 (n=22)	2.31 ± 4.68 (n=70)	**0.072**	3.09 ± 5.76 (n=6)	2.65 ± 4.92 (n=90)	0.691
Number of neutrophile before surgery (×10^3^)*	8.81 ± 3.65 (n=1)	7.08 ± 2.69 (n=14)	0.196	7.08 ± 2.77 (n=3)	7.05 ± 2.72 (n=8)	0.829	7.08 ± 2.72 (n=2)	7.16 ± 2.84 (n=11)	0.875
Δ Number of neutrophiles (×10^3^)*	4.60 ± 5.89 (n=1)	1.68 ± 3.83 (n=109)	**0.031**	2.32 ± 3.70 (n=22)	1.63 ± 3.89 (n=70)	0.277	1.95 ± 3.61 (n=5)	1.65 ± 4.04 (n=89)	0.672
Number of lymphocytes before surgery (×10^3^)*	1.51 ± 0.62 (n=1)	1.44 ± 0.64 (n=9)	0.757	1.47 ± 0.69 (n=1)	1.50 ± 0.65 (n=6)	0.601	1.50 ± 0.56 (n=2)	1.45 ± 0.67 (n=7)	0.627
Δ Number of lymphocytes (×10^3^)*	0.12 ± 1.26 (n=1)	− 0.21 ± 0.61 (n=105)	0.130	− 0.24 ± 0.52 (n=21)	− 0.24 ± 0.67 (n=67)	0.843	− 0.26 ± 0.53 (n=6)	− 0.19 ± 0.66 (n=85)	0.509
RDW*	14.94 ± 1.69 (n=0)	14.10 ± 1.79 (n=6)	0.157	14.33 ± 1.76 (n=1)	13.95 ± 1.84 (n=4)	0.149	14.48 ± 2.04 (n=0)	14.05 ± 1.59 (n=5)	0.147
Blood sugar baseline*	185.11 ± 103.71 (n=1)	156.28 ± 73.56 (n=57)	0.432	170.15 ± 84.50 (n=14)	143.76 ± 62.34 (n=33)	**0.028**	161.37 ± 76.41 (n=3)	153.91 ± 72.92 (n=49)	0.596
Surgical factors									
Operation time (min)*	168.33 ± 42.28 (n=1)	184.25 ± 58.08 (n=32)	0.302	186.02 ± 59.40 (n=5)	182.16 ± 55.66 (n=21)	0.543	188.28 ± 73.04 (n=2)	182.07 ± 55.01 (n=27)	0.629
Anesthesia time (min)*	180.00 ± 40.00 (n=0)	194.34 ± 57.32 (n=3)	0.298	193.87 ± 58.19 (n=0)	195.54 ± 56.32 (n=1)	0.958	195.00 ± 69.66 (n=0)	193.89 ± 54.78 (n=2)	0.926
Anesthesia type	(n=0)	(n=2)	0.589	(n=0)	(n=1)	0.595	(n=0)	(n=0)	0.847
Spinal	9 (90%)	227 (76.2%)	–	75 (80.6%)	111 (74.5%)	–	29 (76.3%)	175 (75.4%)	–
General	1 (10%)	67 (22.5%)	–	16 (17.2%)	36 (24.2%)	–	9 (23.7)	55 (23.7%)	–
Spinal & general	0 (0%)	4 (1.3%)	–	2 (2.2%)	2 (1.3%)	–	0 (0%)	2 (.9%)	–
Surgical technique	(n=0)	(n=2)	**0.077**	(n=1)	(n=0)	0.749	(n=38)	(n=1)	0.463
DHS	6 (60.0%)	251 (84.2%)	–	79 (85.9%)	125 (83.3%)	–	31 (81.6%)	194 (84.0%)	–
Arthroplasty	1 (10.0%)	8 (2.7%)	–	2 (2.2%)	4 (2.7%)	–	1 (2.6%)	4 (1.7%)	–
Nail	2 (20.0%)	27 (9.1%)	–	8 (8.7%)	15 (10%)	–	6 (15.8%)	21 (9.1%)	–
DCS	1 (10.0%)	4 (1.3%)	–	0 (0%)	3 (2%)	–	0 (0%)	5 (2.2%)	–
DHS + anti-rotation	0 (0%)	8 (2.7%)	–	3 (3.3%)	3 (2%)	–	0 (0%)	7 (3.0%)	–
Blood transfusion	8 (80%), (n=0)	108 (36.2%), (n=2)	**0.007**	49 (52.7%), (n=0)	42 (28%), (n=0)	**0.000**	23 (60.5%), (n=0)	78 (33.8%), (n=1)	**0.002**

^*^Given as the mean and standard deviation.

Significant values are in bold.

Patients who died in hospital tend to have higher white blood cells before surgery (p = 0.024), BUN before surgery (p = 0.000), an increased Δ White blood cells (p = 0.033), Δ Cr (mg/dL) (p = 0.077), Δ Neutrophile/Platelet (p = 0.000), Δ Number of Neutrophiles (p = 0.031), and a significant drop in platelet count (p = 0.021).

Patients experiencing postoperative complications in the hospital were more likely to have an increased Δ Cr (p = 0.017), Δ BUN (p = 0.099), and Na before surgery (p = 0.022). Patients who died in the long term were more likely to be female (p=0.013), and those with a lower rate of smoking (p=0.008), a lower Hemoglobin before surgery (p=0.000), a lower drop in hemoglobin (p=0.020), longer Duration of admission to surgery (P=0.004), to have Diabetes Mellitus (p=0.004), BUN before surgery (p=0.000), K before surgery (p=0.076), Δ Na (p=0.064), Δ Neutrophile/Lymphocyte (p=0.072), and Blood sugar baseline (p=0.028). Older age, history of HTN or IHD, blood transfusion (before or after surgery), and higher creatine levels before surgery lead to the worse outcome (in hospital mortality, long-term mortality or in hospital complication).

**Figure 1:**
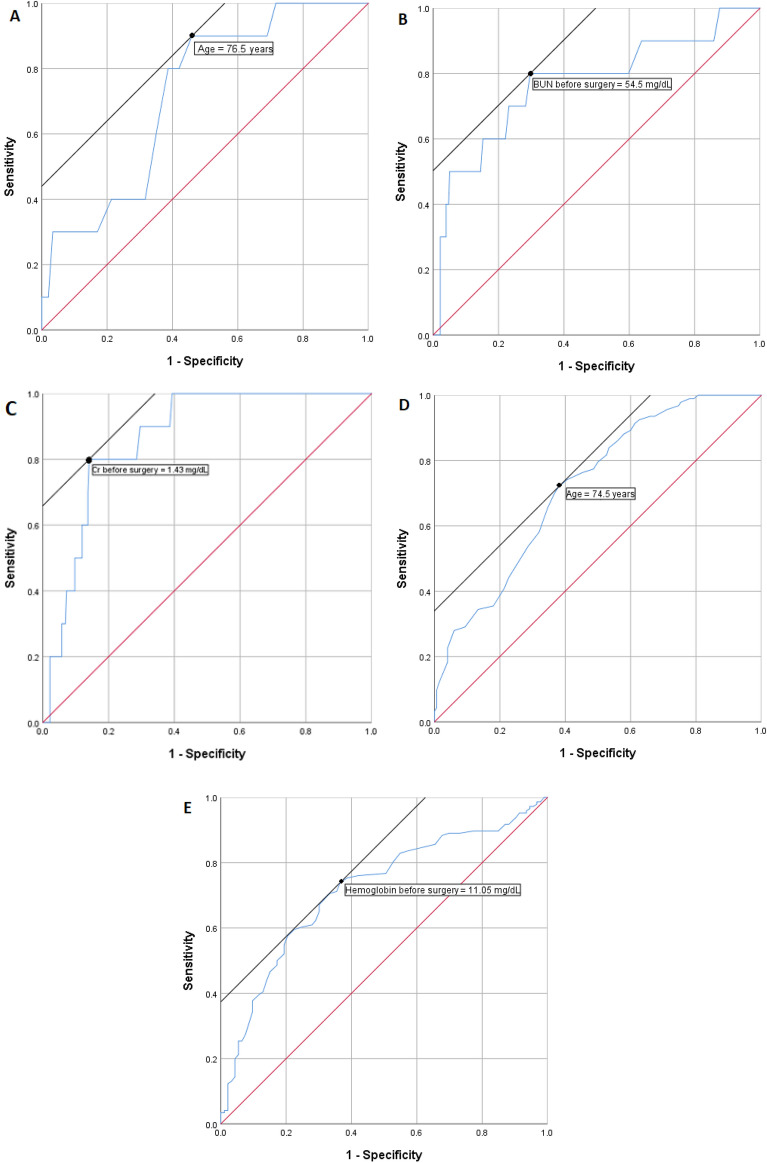
Receiver operating characteristics curves (ROC) for Age (**A**), BUN before surgery (**B**), and Cr before surgery (**C**) while those who die in hospital are considered the positive result of the test. ROC for age (**D**) Hemoglobin before surgery (**E**) while those who die in long term and those who remain alive are considered the positive result of the test respectively.

**Figure 2:**
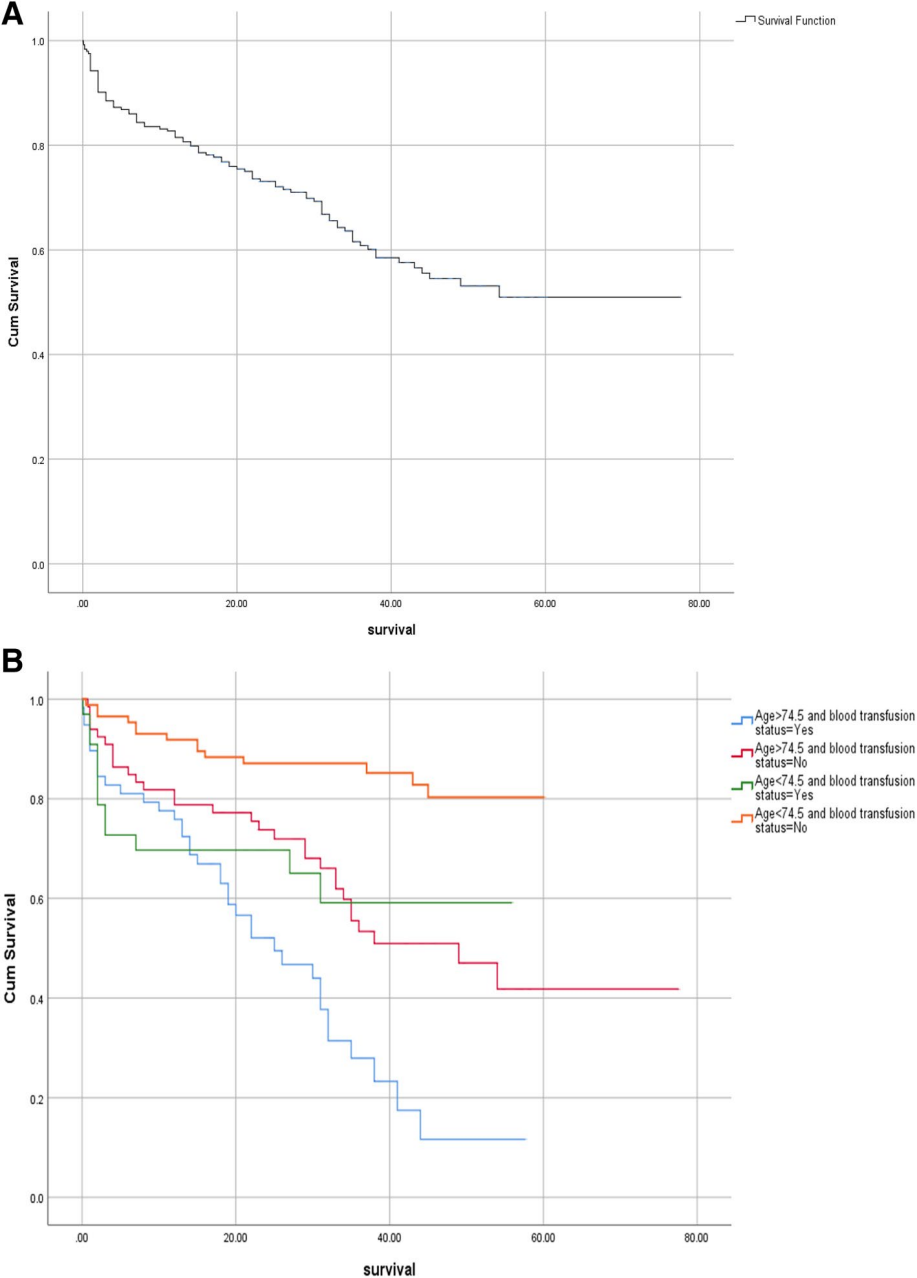
Death-free survival analysis. Total population (first) and stratified population (second).

To develop a regression model for in-hospital mortality as the dependent variable, Δ White blood cell, Δ Cr, and Δ Neutrophile/Platelet were excluded due to high interaction with other variables (The variables which measured the same marker before surgery and is significantly different between groups). The p-value of the Hosmer and Lameshow test is 0.998. Δ Number of Neutrophiles is significant in multivariate analysis. The result is shown in Table 3. ROC analysis was performed for quantitative variables correlated with in-hospital mortality in univariate analysis. The AUC for age (0.721, 95% CI [0.586–0.856]), BUN before surgery (0.770, 95% CI [0.596–0.943]), and Cr before surgery (0.866, 95% CI [0.790–0.941]) were more than 0.70. (Fig. 2). The optimal cut-off values for age, BUN before surgery, and Cr before surgery were 78.5 years (sensitivity = 0.900 and specificity = 0.540), 54.5 mg/dL (sensitivity = 0.800 and specificity = 0.703), and 1.43 mg/dL (sensitivity = 0.800 and specificity = 0.859). Age, HTN, IHD, Cr before surgery, Na before surgery, Δ BUN, and blood transfusion are included in the regression model for in-hospital complications. The p-value of the Hosmer and Lameshow test is 0.117. Na before surgery is the main determinant. The model is explained in Table 4. The AUC for age, Cr before surgery, Na before surgery, Δ BUN were all less than 0.70. The predictive model for long-term mortality was obtained by cox regression and ΔHb is excluded due to interaction with hemoglobin before surgery; ΔNa and Δ Neutrophile/lymphocyte were also excluded to reach the fittest model available. Age and blood transfusion are the main determinants. The model is explained in Table 5. The AUC for age (0.720, 95% CI [0.657–0.783]) and Hemoglobin before surgery (0.718, 95% CI [0.652–0.784] were more than 0.70. the optimal cut-off value for age and Hemoglobin before surgery were 74.5 years (sensitivity = 0.72 and specificity = 0.62) and 11.05 mg/dL (sensitivity = 0.74 and specificity = 0.634) (Fig. 1).

**Table 3:** Binary logistic regression of included variables and in-hospital mortality as the dependent variable.

	B	S.E.	Sig.	Exp(B)	95% CI for EXP(B)	
					Lower	Upper
Age	0.114	0.062	0.065	1.121	0.993	1.266
HTN	0.321	1.343	0.811	1.379	0.099	19.166
IHD	1.721	1.226	0.161	5.588	0.505	61.832
Cr before surgery	1.162	0.694	0.094	3.197	0.82	12.468
BUN before surgery	− 0.001	0.018	0.945	0.999	0.965	1.034
Surgical technique	0.472	0.32	0.14	1.603	0.856	2.999
Blood transfusion	0.563	1.025	0.583	1.756	0.236	13.095
ΔNumber of neutrophiles	**0.25**	**0.124**	**0.044**	**1.284**	**1.006**	**1.637**
ΔPlatelet	− 0.016	0.008	0.052	0.984	0.968	1
WBC before surgery	0.146	0.105	0.164	1.158	0.942	1.423
Constant	− 18.206	6.201	0.003	0		

Significant values are in bold.

**Table 4:** Binary logistic regression of included variables and in-hospital complications as the dependent variable.

	B	S.E.	Sig.	Exp(B)	95% CI for EXP(B)	
					Lower	Upper
Age	0.037	0.025	0.141	1.037	0.988	1.089
HTN	0.046	0.533	0.932	1.047	0.368	2.976
IHD	− 0.341	0.548	0.534	0.711	0.243	2.08
Cr before surgery	0.235	0.301	0.436	1.264	0.701	2.282
Δ BUN	0.013	0.008	0.113	1.013	0.997	1.029
Na before surgery	**0.151**	**0.059**	**0.011**	**1.163**	**1.035**	**1.306**
Blood transfusion	0.714	0.473	0.131	2.043	0.809	5.161
Constant	− 25.841	8.629	0.003	0		

Significant values are in bold.

**Table 5:** Cox regression of included variables and long-term mortality as the dependent variable.

	B	SE	Sig.	Exp(B)	95.0% CI for Exp(B*)*	
					Lower	Upper
Sex	− 0.195	0.379	0.607	0.823	0.392	1.73
Age	**0.051**	**0.015**	**0.001**	**1.052**	**1.021**	**1.084**
Smoke	0.19	0.42	0.65	1.21	0.531	2.756
Height	− 0.012	0.02	0.549	0.988	0.95	1.028
Duration of admission to surgery	0.032	0.032	0.309	1.033	0.971	1.099
HTN	− 0.067	0.336	0.841	0.935	0.484	1.805
IHD	0.248	0.35	0.479	1.281	0.645	2.546
DM	0.487	0.388	0.209	1.628	0.761	3.48
Hb before surgery	− 0.123	0.094	0.192	0.884	0.735	1.064
Cr before surgery	0.146	0.26	0.573	1.158	0.696	1.927
BUN before surgery	0.009	0.005	0.094	1.009	0.998	1.02
K before surgery	− 0.631	0.325	0.052	0.532	0.282	1.006
Blood sugar baseline	0.001	0.002	0.611	1.001	0.997	1.006
Blood transfusion	**0.659**	**0.324**	**0.042**	**1.932**	**1.023**	**3.648**

Significant values are in bold.

Patients stratified into 4 groups base on blood transfusion status and age. Kaplan–Meier survival curves of four groups are demonstrated. The 54 months survival of total population is 0.51 (SE=0.044) (Fig. 2).

## Discussion

The results of our study suggest that a significant rise in number of neutrophile may be associated with in-hospital mortality. Those with increased Na before surgery are more likely to experience in hospital complication. Age is the main determinant of long-term mortality alongside with intra and post-operative blood transfusion.

Post-op neutrophil as a biomarker representing infection was correlated with short-term mortality^19^. Neutrophile count was positively correlated with size of infarction, and Ischemic and non-ischemic heart failure are associated with increased innate leukocytes, and post-op heart failure has a robust association with mortality after hip fracture^19–21^. After stroke neutrophil start to degrade blood brain barrier and predispose brain to a second injury and by several mechanism worsens outcome^22^. Furthermore, in acute ischemic strokes, peripheral neutrophil counts are correlated with larger infarct volumes and fatal outcomes^23^. In hypertensive population neutrophil count increase the risk of first stroke and stroke is one of the post-op comorbidities which increase the risk of mortality in those with hip fracture^19,24^.

In a cohort study of Asian population, 14,744 elderly patients with hip fracture were followed up for 11 years. 10973 patients included in the transfusion group and the adjusted relative risk of mortality was 1.64, 1.58, 1.43 for 90 days, 180 days, and 1 year respectively^25^. In our study the adjusted odds ratio of mortality was 1.932 (95% CI [1.023–3.648], p=0.042). It is believed that there might be immunosuppressive consequences with blood transfusion by suppressing CD3 (T-lymphocytes)^26^. This could result in making patients susceptible to infection which is supported by a meta-analysis of 20 studies which reported an odds ratio of 5.263 (range, 5.03–5.43) for bacterial infection in trauma patients while infection is a risk factor of long-term mortality in the study of Roche et al.^19,27,28^. A large blood transfusion may lead to fluid overload in elderly who are small and frail. Comorbidities like HTN, chronic kidney disease, and previous heart failure as predisposing factor in combination with large blood transfusion may lead to iatrogenic heart failure and heart failure is the most important risk factor of long-term mortality after hip fracture^19,29^. To overcome this problem other blood product including iron supplements, Erythropoietin, or anti-fibrinolytics should be considered^30–32^. However, in a meta-analysis of 54 studies in 2015 the results don’t demonstrate an increased risk of long-term mortality in those with blood transfusion after adjusting for all comorbidities^33^. Further prospective studies with larger sample size are needed to clarify the effect of blood transfusion on long-term mortality. In our study 93 patients (38%) died in long-term and based on Kaplan-Meier analysis the 54-month survival of our patients is 51% and one-year mortality is nearly 15%. Another study by Mehdi Nasab et al. reported a 5-year mortality rate of 37% and a one-year mortality rate of 21%, but this study calculated the mortality rate by dividing the number of deaths in five years by the total population^34^. A randomized clinical trial by Moradi et al. reported a higher one-year mortality rate of 21% compared to our study^35^. In a systematic review and meta-analysis by Ma et al. the rate of early mortality following intertrochanteric fracture was 15.1%^36^. The in-hospital mortality rate reported in the literature ranged from 1.2 to 1.8%, which is lower than the mortality rate of our study (3.23%)^8,37,38^. It is worth noting that our hospital is a referral center, and our patients mainly come from regions with poor economic and sanitary conditions.

Our study found that higher levels of Na are associated with an increased risk for complications in hospital. Dehydration caused by water loss is best diagnosed by serum osmolality in older people^39^. Dehydration is a major problem in the geriatrics with hip fractures. In a retrospective cohort study in 2015 the application of preoperative hemodynamic preconditioning protocol (PHP) results in lower complications for patients with hip fracture. Patients with hip fractures who were deemed at high risk for complications or mortality were treated following the PHP protocol to ensure adequate perfusion and oxygenation and to optimize hemodynamics before surgery^40^. In the study by Lindholm et al. dehydration was reported as a prognostic factor for pressure ulcers at discharge for those with hip fracture (p=0.005), however, we had only two cases of pressure ulcers^41^. In a study of 45 patients following hip fracture surgery, dehydration increased the chances of complications by nearly four times (P<0.015); Dehydrated patients presented with confusion, desaturation requiring oxygen treatment, and cardiovascular problems^42^. Our results are in contrast with a study of 8719 patients with total hip arthroplasty in which dehydration didn’t show any significant relationship with 30-day complications and appears as a protective factor for 30-day readmission (P=0.001). The main difference of last study and our study is the acute setting of present study. Anemia at presentation is risk factor for 30-day readmission and those with dehydration are usually considered as non-anemic group^14^. One of the reasons could be the blood transfusion in anemic group in the acute setting of hip fracture which increases the infection after surgery while in the elective setting of arthroplasty administration of TXA reduces the risk of readmission^14,43^.

Several limitations of study should be mentioned. The reliability and accuracy of AO/OTA classification is questionable^44^. Distribution of cases in subgroups of AO/OTA, type of implant, and type of anesthesia was unbalanced and this leads to random error. The retrospective nature of study which was conducted in one center result in selection bias. Unfortunately, because of recall bias we were not able to analyze the cause of death. The complication was an outcome with high heterogeneity which cannot be sub grouped due to unbalanced distribution of type of complication. Finally, we were not able to introduce a comorbidity index into our analysis.

## Conclusion

Among different analytical factors Na before surgery as a biomarker presenting dehydration was the main prognostic factor for in hospital complications. In hospital mortality was mainly because of infection and long-term mortality was associated with blood transfusion.

## Data Availability

All data generated or analyzed during this study are included in this published article.

## Abbreviations

BMI: Body mass index
ROC: Receiver operating characteristic
AUC: Area under the ROC curve
Cr: Creatine
BUN: Blood urea nitrogen
HTN: Hypertension
IHD: Ischemic heart disease
Hb: Hemoglobin
DM: Diabetes mellitus

## References

[1] Gullberg B, Johnell O, Kanis JA (1997). World-wide projections for hip fracture. Osteoporos. Int..

[2] Adeyemi A, Delhougne G (2019). Incidence and economic burden of intertrochanteric fracture: A medicare claims database analysis. JBJS Open Access.

[3] Nazrun AS, Tzar MN, Mokhtar SA, Mohamed IN (2014). A systematic review of the outcomes of osteoporotic fracture patients after hospital discharge: Morbidity, subsequent fractures, and mortality. Ther. Clin. Risk Manag..

[4] Mundi S, Pindiprolu B, Simunovic N, Bhandari M (2014). Similar mortality rates in hip fracture patients over the past 31 years. Acta Orthop..

[5] Belmont PJ, Garcia EJ, Romano D, Bader JO, Nelson KJ, Schoenfeld AJ (2014). Risk factors for complications and in-hospital mortality following hip fractures: A study using the National Trauma Data Bank. Arch. Orthop. Trauma Surg..

[6] Biçen Ç, Akdemir M, Türken MA, Çekok K, Ekin A, Turan AC (2021). Analysis of risk factors affecting mortality in elderly patients operated on for hip fractures: A retrospective comparative study. Acta Orthop. Traumatol. Turc..

[7] Jiang L, Chou ACC, Nadkarni N, Ng CEQ, Chong YS, Howe TS (2018). Charlson comorbidity index predicts 5-year survivorship of surgically treated hip fracture patients. Geriatr. Orthop. Surg. Rehabil..

[8] Pinto IP, Ferres LFB, Boni G, Falótico GG, Moraes M, Puertas EB (2019). Does early surgical fixation of proximal femoral fractures in elderly patients affect mortality rates?. Rev. Bras. Ortop. (Sao Paulo).

[9] Akinleye SD, Garofolo G, Culbertson MD, Homel P, Erez O (2018). The role of BMI in hip fracture surgery. Geriatr. Orthop. Surg. Rehabil..

[10] Cha YH, Lee YK, Koo KH, Wi C, Lee KH (2019). Difference in mortality rate by type of anticoagulant in elderly patients with cardiovascular disease after hip fractures. Clin. Orthop. Surg..

[11] Harty JA, McKenna P, Moloney D, D'Souza L, Masterson E (2007). Anti-platelet agents and surgical delay in elderly patients with hip fractures. J. Orthop. Surg. (Hong Kong).

[12] Maheshwari R, Acharya M, Monda M, Pandey R (2011). Factors influencing mortality in patients on antiplatelet agents presenting with proximal femoral fractures. J. Orthop. Surg. (Hong Kong).

[13] Zhao P, Lian X, Dou X, Bi Q, Quan R, Tong P (2015). Intertrochanteric hip fracture surgery in Chinese: Risk factors for predicting mortality. Int. J. Clin. Exp. Med..

[14] Ryan G, Nowak L, Melo L, Ward S, Atrey A, Schemitsch EH (2020). Anemia at presentation predicts acute mortality and need for readmission following geriatric hip fracture. JBJS Open Access.

[15] Lu J, Chen YY, Zhang L, Li YG, Wang C (2016). Laboratory nutritional parameters predict one-year mortality in elderly patients with intertrochanteric fracture. Asia Pac. J. Clin. Nutr..

[16] Borges A, Torres J, São Simão R, Cabral AT, Pinto R (2014). Impact of preoperative analytical values on post-operative mortality rate of intertrochanteric fractures. Acta Med. Port..

[17] Demirel E, Şahin A (2021). Predictive value of blood parameters and comorbidities on three-month mortality in elderly patients with hip fracture. Cureus.

[18] Youden WJ (1950). Index for rating diagnostic tests. Cancer.

[19] Roche JJ, Wenn RT, Sahota O, Moran CG (2005). Effect of comorbidities and postoperative complications on mortality after hip fracture in elderly people: Prospective observational cohort study. BMJ.

[20] Kain V, Halade GV (2020). Role of neutrophils in ischemic heart failure. Pharmacol. Ther..

[21] Chia S, Nagurney JT, Brown DF, Raffel OC, Bamberg F, Senatore F (2009). Association of leukocyte and neutrophil counts with infarct size, left ventricular function and outcomes after percutaneous coronary intervention for ST-elevation myocardial infarction. Am. J. Cardiol..

[22] Hermann DM, Kleinschnitz C, Gunzer M (2018). Implications of polymorphonuclear neutrophils for ischemic stroke and intracerebral hemorrhage: Predictive value, pathophysiological consequences and utility as therapeutic target. J. Neuroimmunol..

[23] Buck BH, Liebeskind DS, Saver JL, Bang OY, Yun SW, Starkman S (2008). Early neutrophilia is associated with volume of ischemic tissue in acute stroke. Stroke.

[24] Zhang Z, Zhou C, Liu M, Zhang Y, Li H, He P (2021). Neutrophil counts and the risk of first stroke in general hypertensive adults. Hypertens. Res..

[25] Jang SY, Cha YH, Yoo JI, Oh T, Kim JT, Park CH, Choy WS, Ha YC, Koo KH (2020). Blood transfusion for elderly patients with hip fracture: A Nationwide Cohort Study. J. Korean Med. Sci..

[26] Sousa R, Salinas JC, Navarro M, Güemes A, Torcal J, García-Alvarez F (2000). Autologous blood transfusion as an immunomodulator in experimental sepsis. Int. J. Surg. Investig..

[27] Hill GE, Frawley WH, Griffith KE, Forestner JE, Minei JP (2003). Allogeneic blood transfusion increases the risk of postoperative bacterial infection: A meta-analysis. J. Trauma.

[28] Blumberg N, Triulzi DJ, Heal JM (1990). Transfusion-induced immunomodulation and its clinical consequences. Transfus. Med. Rev..

[29] Tran P, Banerjee P (2020). Iatrogenic decompensated heart failure. Curr. Heart Fail. Rep..

[30] Lee H, Yuh Y (2018). A paradigm shift: Perioperative iron and erythropoietin therapy for patient blood management. Hanyang Med. Rev..

[31] Voorn V, Hout A, So-Osman C, Vlieland T, Nelissen R, Van den Akker E (2016). Erythropoietin to reduce allogeneic red blood cell transfusion in patients undergoing total hip or knee arthroplasty. Vox Sang..

[32] Guzel Y, Gurcan OT, Golge UH, Dulgeroglu TC, Metineren H (2016). Topical tranexamic acid versus autotransfusion after total knee arthroplasty. J. Orthop. Surg. (Hong Kong).

[33] Potter LJ, Doleman B, Moppett IK (2015). A systematic review of pre-operative anaemia and blood transfusion in patients with fractured hips. Anaesthesia.

[34] Nasab SAM, Khorramdin E (2017). The assessment of mortality and quality of life after intertrochanteric fracture of femur in patients older than 60 at Emam Khomeini Hospital of Ahvaz. Pak. J. Med. Sci..

[35] Moradi A, Moradi M, Emadzadeh M, Bagheri F (2021). Comparison of the dynamic hip screw with the dynamic hip external fixator for intertrochanteric fractures: Report of a randomized controlled trial. Arch. Bone Jt. Surg..

[36] Ma J, Xing D, Ma X, Xu W, Wang J, Chen Y, Song D (2012). The percutaneous compression plate versus the dynamic hip screw for treatment of intertrochanteric hip fractures: A systematic review and meta-analysis of comparative studies. Orthop. Traumatol. Surg. Res..

[37] Kiriakopoulos E, McCormick F, Nwachukwu BU, Erickson BJ, Caravella J (2017). In-hospital mortality risk of intertrochanteric hip fractures: A comprehensive review of the US Medicare database from 2005 to 2010. Musculoskelet. Surg..

[38] McHugh MA, Wilson JL, Schaffer NE, Olsen EC, Perdue A, Ahn J, Hake ME (2023). Preoperative comorbidities associated with early mortality in hip fracture patients: A multicenter study. J. Am. Acad. Orthop. Surg..

[39] Hooper L, Bunn D, Jimoh FO, Fairweather-Tait SJ (2014). Water-loss dehydration and aging. Mech. Ageing Dev..

[40] Kusen JQ, van der Vet PCR, Wijdicks FJG, Link BC, Poblete B, van der Velde D, Babst R, Beeres FJP (2021). Does preoperative hemodynamic preconditioning improve morbidity and mortality after traumatic hip fracture in geriatric patients? A retrospective cohort study. Arch. Orthop. Trauma Surg..

[41] Lindholm C, Sterner E, Romanelli M, Pina E, Torra y Bou J, Hietanen H (2008). Hip fracture and pressure ulcers—The Pan-European Pressure Ulcer Study - intrinsic and extrinsic risk factors. Int. Wound J..

[42] Ylinenvaara SI, Elisson O, Berg K, Zdolsek JH, Krook H, Hahn RG (2014). Preoperative urine-specific gravity and the incidence of complications after hip fracture surgery: A prospective, observational study. Eur. J. Anaesthesiol..

[43] Morrison RJM, Tsang B, Fishley W, Harper I, Joseph JC, Reed MR (2017). Dose optimisation of intravenous tranexamic acid for elective hip and knee arthroplasty: The effectiveness of a single pre-operative dose. Bone Joint Res..

[44] Chan G, Hughes K, Barakat A, Edres K, da Assuncao R, Page P (2021). Inter- and intra-observer reliability of the new AO/OTA classification of proximal femur fractures. Injury.

